# Expanding relationship science to unpartnered singles: What predicts life satisfaction?

**DOI:** 10.3389/fpsyg.2022.904848

**Published:** 2022-09-16

**Authors:** Lisa C. Walsh, Ariana M. Gonzales, Lucy Shen, Anthony Rodriguez, Victor A. Kaufman

**Affiliations:** ^1^Department of Psychology, University of California, Los Angeles, Los Angeles, CA, United States; ^2^RAND Corporation, Boston, MA, United States

**Keywords:** singles, life satisfaction, well-being, relationships, self-esteem, personality

## Abstract

Singles are an understudied yet growing segment of the adult population. The current study aims to expand the lens of relationship science by examining the well-being of unpartnered, single adults using latent profile analysis. We recruited singles (*N* = 4,835) closely matched to the United States census (ages 18-65; 57.5% female; 71.1% White; 14.5% Black; 13.8% Hispanic) for an exploratory cross-sectional survey using five variables that strongly predict well-being (friendship satisfaction, family satisfaction, self-esteem, neuroticism, and extraversion). All five variables significantly predicted life satisfaction for the full sample. Latent profile analyses detected 10 groups (or profiles) of singles. Half of the profiles were happy (above the full sample mean of life satisfaction) and half of the profiles were unhappy (below the mean). Each profile had its own unique patterns relating to personal relationships, self-esteem, and personality traits. The happiest profile had the best relationships, self-esteem, and personality, while the unhappiest profile had the worst relationships, self-esteem, and personality. The profiles in between these two extremes had more nuanced patterns. For example, one relatively happy profile in the middle had high friendship satisfaction but low family satisfaction, while an adjacent profile showed the opposite pattern. Overall, singles who had positive relationships—both with themselves and others—were happiest.

## Introduction

“No man is an island, entire of itself; every man is a piece of the continent, a part of the main …” —John Donne

“Singles studies? What’s that? It is an area of studies that should exist but does not.” —Bella DePaulo

Relationship science has historically focused predominantly on couple (i.e., married or partnered) and parent-child relationships (Berscheid, 1999). Unpartnered singles—often treated as islands unto themselves—are an understudied and growing segment of the adult population that should be better understood (DePaulo, 2014; Fry and Parker, 2021). Although such singles do not have romantic relationships (i.e., they are not married or living with a romantic partner), they do have a variety of other important relationships, including those with close friends and family members.

When researchers study unpartnered singles, such work tends to either report their demographics or compares them to partnered people (e.g., Coombs, 1991; Brown, 2020). Partnered vs. unpartnered comparison studies find that, on average, singles are significantly less happy than those in long-term relationships (Stutzer and Frey, 2006). However, this may be an oversimplification, and singles may differ from each other in unique ways. Solely assessing averages via variable-centered analyses may obscure nuanced trends among singles that may be uncovered by person-centered analyses. In other words, some singles may be very happy, while some may be very unhappy, and others may fall somewhere in between. In the present study, we aimed to use person-centered latent profile analysis to examine the distinct variability of singles’ personal relationships, self-esteem, and personality traits, as well as how combinations of these variables differentiate singles’ happiness levels. Towards this goal, we used five variables to create such profiles: friendship satisfaction, family satisfaction, self-esteem, neuroticism, and extraversion. We chose these variables because they are among the strongest predictors of well-being (Lyubomirsky et al., 2005; Margolis et al., 2021). Below we address relevant issues and research, including why study singles, partnered vs. unpartnered comparison studies, the usefulness of latent profile analysis, and our primary variables of interest.

## Why study singles?

Singles represent a growing subset of the population worthy of study in their own right. As of 2019, 38% of adults ages 25 to 54 in the United States are unpartnered, which is up from 29% in 1990 (Brown, 2020; Fry and Parker, 2021). The share of single adults also tends to vary by demographic subgroup. Although men and women are equally likely to be single, younger and older people (ages 18-29 and 65+) are more likely to be single than those in mid-life (ages 30-64). Black adults are more likely to be single than White or Hispanic adults.

The United States is a highly pro-marriage society where “singlism” (stigma and discrimination directed at the unmarried) is common (Byrne and Carr, 2005; DePaulo, 2014). Recent work suggests Americans believe prejudice against singles is more acceptable than prejudice against other groups, including foreign, gay, lesbian, and bisexual people (Fisher and Sakaluk, 2020). When asked to list characteristics that come to mind, people are more likely to describe those who are married as mature, stable, honest, happy, kind, and loving, and describe those who are unmarried as immature, insecure, self-centered, unhappy, lonely, and ugly (DePaulo and Morris, 2006). These negative stereotypes are even more pronounced when the singles described are older (e.g., 40 vs. 25), yet such stereotypes are usually inaccurate. Negative views about single people also affect partnered people, who may stay in unsatisfying romantic relationships merely because they fear being single (Spielmann et al., 2013).

Besides being largely WEIRD (Western, Educated, Industrialized, Rich, and Democratic), relationship science has also predominantly focused on the nuclear family (married couples and their children)—largely neglecting singles (Berscheid, 1999; DePaulo and Morris, 2006; Henrich et al., 2010). According to DePaulo (2014), “we need perspectives grounded in single life for the same reasons that we have needed Women’s Studies programs, Black Studies programs, and so many other programs representing people who have long been marginalized or ignored in academic research” (p. 64). Notably, some researchers have begun to take up this call (Girme et al., 2016; Kislev, 2018; Fisher et al., 2021; Park Y. et al., 2021), but critical gaps in the literature remain.

Singles may have something unique to add to relationship science, precisely because they tend to maintain more diverse social networks than partnered people do (DePaulo and Morris, 2006; Finkel et al., 2014). Relative to married people, singles are more likely to spend time with their parents, siblings, neighbors, and friends at least once per week (Finkel et al., 2014). Perhaps it is time to give other relationships (e.g., with friends and family) their due (DePaulo and Morris, 2006).

Because satisfaction with marriage (a type of close relationship) is one of the strongest predictors of life satisfaction for spouses (Finkel et al., 2014), singles’ satisfaction with their close relationships (friends and family) may predict their life satisfaction (Diener and Seligman, 2002; Lyubomirsky et al., 2005; Eid and Larsen, 2008; Pinker, 2015). Therefore, studying singles may increase knowledge about human relationships, and further diversify the field; not to mention, better help establish the science of “singles studies” that Bella DePaulo (2014) argued should already exist. Such a perspective would shift the focus from the good marriage to the good life, which may not include marriage at all.

## Partnered vs. unpartnered comparison studies

Most relevant psychological studies that sample singles compare their happiness to that of partnered counterparts. Such studies tend to find that single people are less happy than partnered people (Coombs, 1991; Myers and Diener, 1995; Stack and Eshleman, 1998; Stutzer and Frey, 2006); but the effects are relatively small (e.g., β < 0.05, *d* = 0.13, *r* = 0.14) and inconsistent across countries and time points (ranging from *d* = −0.34 in Latvia in 1995 to *d* = 0.60 in Sweden in 1981; Haring-Hidore et al., 1985; Lucas and Dyrenforth, 2005; Purol et al., 2020). A few studies suggest that partnered and unpartnered people may even have similar levels of happiness (Greitemeyer, 2009; Musick and Bumpass, 2012). More recently, studies have revealed new predictors (e.g., attachment anxiety, attachment avoidance) and moderators (e.g., desire for a romantic partner, conflict avoidance goals, individualistic/post-materialistic values) that may impact singles’ happiness levels (Girme et al., 2016; Kislev, 2018, 2020; Pepping and MacDonald, 2019; MacDonald and Park, 2022).

Some researchers argue that partnered vs. unpartnered comparison studies are flawed for several reasons (e.g., singlism biases, self-selection effects) and these flaws artificially inflate the benefits of marriage (Byrne and Carr, 2005; DePaulo, 2014). We argue that comparison studies are also flawed because they rely heavily on averages that merely compare the mean happiness of partnered people to the mean happiness of unpartnered people, without considering the variability around the mean—effectively concealing individual differences. The happiest single may be happier than the unhappiest spouse. Nevertheless, we do not intend to further compare partnered vs. unpartnered people, but rather to examine how unpartnered singles differ from each other.

## Using latent profile analysis to differentiate singles’ happiness levels

One key way singles may differ from each other includes how happy (or unhappy) they are. Previous comparison studies usually rely exclusively on *variable-centered* analyses (e.g., multiple regression, confirmatory factor analysis, structural equation modeling) that assume all individuals in a given sample come from a single population with one set of averaged parameters (Morin et al., 2016). Yet one mean value cannot accurately summarize everyone’s unique experience. As such, researchers are increasingly turning to *person-centered* analyses (e.g., latent profile analysis, cluster analysis, growth mixture analysis) to examine whether a given sample includes multiple subpopulations that each have their own distinct set of parameters. No approach is necessarily “better” than the other, but each approach can be useful depending on the research question (Howard and Hoffman, 2018). By focusing on person-centered approaches in the present study we hope to yield greater insight into the lives of singles.

For our purposes, latent profile analysis presents a particularly promising avenue to explore the variability of singles’ characteristics and experiences. This statistical tool is used to discover hidden groups in data by determining the probability that participants belong to different groups (or profiles; Ferguson et al., 2019). It is grounded in three main arguments: (1) important individual differences exist, (2) these individual differences occur in a logical way that can be observed via patterns, and (3) some patterns are meaningful and occur across individuals. Latent profile analysis allows researchers to determine how many profiles exist in a population using formal testable models. By using continuous variables known to predict well-being to construct distinct profiles of singles, we can examine how these variables work together to create varying levels of well-being.

To our knowledge, the present study is one of a handful to use latent profile analysis (or other person-centered techniques like cluster analysis) to directly study single adults. Although unpartnered people have been included in similar analyses previously, they usually are not the primary focus (Purol et al., 2020; Park J. H. et al., 2021). However, previous research has used similar techniques to study spouses. Although variable-centered studies initially concluded that, on average, marital quality declines over time for couples, person-centered studies showed that some marriages start high (or low) and stay there, while others decline rapidly (Anderson et al., 2010; Lavner and Bradbury, 2010; Proulx et al., 2017). Thus, a singles-focused latent profile analysis may produce similar novel findings that have been previously overlooked.

## Subjective well-being and its predictors

If we want to determine whether singles differ from each other in latent profile analysis, how should well-being be assessed, and what variables predict its variability? What variables should we use to create unique and meaningful profiles of singles?

### Subjective well-being

Diener and his colleagues Diener (1984), Diener et al. (1999) defined subjective well-being as being comprised of *positive and negative affect* that measure pleasant and unpleasant emotions, *life satisfaction* that evaluates overall quality of life, and *domain satisfaction* that measures satisfaction in specific domains such as work, finances, and health. Because the affective components of subjective well-being (positive and negative emotions) tend to be more transient—changing rapidly in response to daily life events—we assessed unpartnered singles’ happiness with the two relatively more stable cognitive components: life satisfaction and domain satisfaction. However, we use the terms “well-being,” “life satisfaction,” and “happiness” interchangeably, as researchers have done in the past (e.g., Welsch, 2006; Christoph, 2010). We now review research on our selected predictors of well-being.

### Personal relationships

Humans are an “ultra-social” species dependent on positive relationships with others (Fredrickson, 2001; Tomasello, 2014). Self-determination theory posits that *relatedness* is one of three basic psychological needs (Deci and Ryan, 2012), and research shows that social connection (i.e., closeness, belonging) is critical to human health and happiness (Lyubomirsky et al., 2005; Eid and Larsen, 2008; Ng et al., 2012; Pinker, 2015; Holt-Lunstad et al., 2017). Meta-analytic work also shows that sociality (e.g., marital satisfaction, time spent with friends) is associated with well-being (*r*s = 0.15 to.37; Lyubomirsky et al., 2005; Margolis et al., 2021). Additional research suggests that people with a diverse portfolio of *emotionships*—specific social relationships that serve distinct emotion-regulation needs (e.g., cheering up sadness, soothing anxiety)—have higher well-being (Cheung et al., 2015). People’s strongest social contacts consist of romantic relationships, family relationships, and friendships (Kaufman et al., 2022b). Because single, unpartnered people do not have long-term romantic relationships, we focused on their relationships with friends and family.

#### Friendship satisfaction

Friendships have been described as the defining relationship of the modern era (Vernon, 2005). Researchers have proposed varying definitions, but most describe friendships as personal relationships that are voluntary, mutual, and enjoyable (Kaufman, 2020). Importantly, people with satisfying, close friendships are happier and healthier. Recently, researchers have used bifactor modeling and item response theory to develop a friendship satisfaction scale, and found it was positively associated with well-being (*r*s = 0.38 to.44; Kaufman et al., 2022a).

#### Family satisfaction

In contrast, family (e.g., parents, children, siblings) involve relatively life-long, less voluntary relationships, because people are ethically, legally, and biologically bound to their kin (Fuller-Iglesias et al., 2015). Accordingly, the associations between family and well-being are more complicated—sometimes positive, other times negative. Although family members comprise many of our closest social networks and support us during stressful life events (Birditt and Antonucci, 2007), they are also sources of sustained conflict and adverse childhood experiences such as emotional, physical, or sexual abuse (Antonucci et al., 2011; Giovanelli et al., 2016).

### Self-esteem

A common view posits that self-esteem and happiness are so innately linked that it is difficult to separate them (Lyubomirsky et al., 2006). In support of this view, self-esteem and happiness are moderately correlated (rs = 0.31 to.59; Lyubomirsky and Lepper, 1999; Lyubomirsky et al., 2006; Margolis et al., 2021; Schimmack and Diener, 2003). However, Lyubomirsky et al. (2006) examined the similarities and differences among the two constructs with confirmatory factor analysis and concluded that happiness and self-esteem are indeed distinct constructs. They also found that extraversion, neuroticism, and social relationships (e.g., friendship satisfaction, lack of loneliness) best predicted happiness, while optimism and lack of hopelessness best predicted self-esteem.

### Personality

We also focused on the two personality traits that are most related to subjective well-being: neuroticism and extraversion (Steel et al., 2008; Lucas, 2018; Margolis et al., 2021).

#### Extraversion

The trait of extraversion is composed of three facets (sociability, assertiveness, and energy level; Soto and John, 2017). To assess it, individuals usually rate how much they view themselves as someone who is outgoing, sociable, assertive, and full of energy. Numerous studies find strong positive associations between extraversion and well-being (meta-analytic *r*s = 0.37 to.44; Costa and McCrae, 1980; DeNeve and Cooper, 1998; Steel et al., 2008; Lucas, 2018; Anglim et al., 2020; Margolis et al., 2021).

#### Neuroticism

The trait of neuroticism is also composed of three facets (anxiety, depression, and emotional volatility; Soto and John, 2017). Measures of neuroticism typically ask individuals to rate how much they view themselves as someone who often worries a lot, feels sad, and has mood swings. A wide variety of research finds strong negative associations between neuroticism and well-being (meta-analytic *r* = −0.46; Costa and McCrae, 1980; DeNeve and Cooper, 1998; Lucas, 2018; Anglim et al., 2020; Margolis et al., 2021).

### Other variables that predict well-being

What about other variables associated with well-being? Meta-analytic investigations have identified countless additional factors, including meaning in life (*r* = 0.47), self-compassion (*r* = 0.47), optimism (*r* = 0.43), and religiosity (*r* = 0.10), among others (Lyubomirsky et al., 2005; Margolis et al., 2021). However, no study can include every variable, so we selected what we believe are the strongest and most cited correlates (personal relationships, self-esteem, and personality). Further, many of the unselected variables (e.g., meaning in life, optimism) likely overlap with our selected variables (e.g., family satisfaction, self-esteem). For example, family and friends are two of the most reported sources of meaning in life (Silver et al., 2021), and optimism is the strongest predictor of self-esteem (Lyubomirsky et al., 2006).

## The present study

Building on the research summarized above, the current exploratory study aims to expand the lens of relationship science by investigating what influences unpartnered singles’ well-being using latent profile analysis. We focused especially on how distinct profiles of singles differ from each other. Our goal was to determine what predicts singles’ happiness and what differentiates happy singles from unhappy singles. We had two primary research questions: (1) How well do our five identified variables (friendship satisfaction, family satisfaction, self-esteem, neuroticism, and extraversion) predict life satisfaction overall? (2) Using the five variables, can we identify similar, more complex profiles (or groups) of people to better characterize the heterogeneity (i.e., distinct array) of single individuals?

## Method

### Study design and participants

In May and June 2021, we recruited participants for a cross-sectional study via professional data insights and research platform, Dynata. People who met demographic criteria matched to the United States census were emailed a study invitation with an online survey link. Only those who indicated they currently had *no* main romantic involvement (e.g., girlfriend, partner, spouse) and were between the ages of 18 and 65 years were qualified to participate. Of those who were qualified, 5,010 completed all survey questions with no missing data. They were compensated accordingly with cash, reward points, or discounts.

To ensure data quality, we randomly inserted engagement checks throughout the survey to monitor whether participants were paying attention (e.g., “Please select ‘Somewhat Agree’ here”). Participants who failed any of seven engagement checks were excluded from the sample. Additionally, participants who straight-lined through four or more scales were also excluded from analyses, yielding a final sample of 4,835 single adults (ages 18-65; *M*_*age*_ = 40.88; 57.5% female; 71.1% White; 14.5% Black; 13.8% Hispanic) with varying lengths of singlehood (1.8% less than one week; 4.3% 1-3 weeks; 7.1% 1-2 months; 11.3% 3-6 months; 8.6% 7-11 months; 66.9% 1 + years). Participant demographics for the full sample, as well as for each profile are reported in Table 1.

**TABLE 1:** Participant demographic characteristics.

Characteristic	Full sample	Profile 1	Profile 2	Profile 3	Profile 4	Profile 5	Profile 6	Profile 7	Profile 8	Profile 9	Profile 10
	
	(*N* = 4835)	(*n* = 670)	(*n* = 912)	(*n* = 716)	(*n* = 142)	(*n* = 152)	(*n* = 149)	(*n* = 1269)	(*n* = 318)	(*n* = 319)	(*n* = 188)
Age (*M* ± *SD* in years)	40.9 (15.3)	43.4 (14.9)	47.2 (14.3)	33.0	50.6	44.9	50.1	38.7	42.6	32.73	39.1
				(12.7)	(12.4)	(15.9)	(13.8)	(14.9)	(14.0)	(13.7)	(14.3)
Sex (% Female)	57.5%	56.4%	55.8%	57.8%	51.4%	70.4%	61.1%	53.1%	63.8%	68.3%	60.1%
Race/Ethnicity*											
White/Caucasian	71.1%	72.2%	74.6%	65.1%	76.1%	71.1%	68.5%	68.6%	73.0%	74.0%	80.9%
Black/African American	14.5%	16%	13.3%	16.3%	15.5%	15.8%	18.8%	13.6%	14.8%	13.8%	9.6%
Hispanic/Latino(a)	13.8%	13.3%	10.4%	18.0%	6.3%	12.5%	13.4%	15.1%	13.2%	13.8%	14.4%
Asian	6.6%	4.0%	5.4%	8.5%	4.2%	6.6%	3.4%	8.8%	5.3%	6.3%	5.9%
Other	2.8%	2.4%	3.7%	1.3%	7.0%	3.3%	4.7%	2.8%	2.5%	2.8%	2.1%
**Education Level**											
Less than high school	1.9%	1.2%	0.8%	1.7%	1.4%	1.3%	3.4%	2.0%	2.5%	4.1%	5.9%
High school graduate	20.1%	17.5%	15.0%	20.1%	25.4%	16.4%	20.1%	20.8%	21.7%	28.8%	31.9%
Some college/vocational	29.6%	28.1%	27.1%	29.3%	32.4%	33.6%	26.8%	29.6%	33.6%	34.5%	29.3%
College graduate	35.2%	34.9%	38.8%	37.7%	28.2%	34.2%	38.9%	35.3%	32.7%	27.9%	27.7%
Post-graduate	13.0%	17.9%	18.2%	11.2%	12.7%	14.5%	10.7%	12.1%	9.1%	4.7%	5.3%
Prefer not to answer	0.1%	0.4%	0.1%	0%	0%	0%	0%	0.2%	0.3%	0%	0%
**Household Income**											
Less than $30,000	23.4%	16.6%	18.6%	17.5%	40.1%	27.6%	38.3%	22.1%	35.8%	32.3%	38.3%
30,000 - $49,999	26.4%	26.1%	27.1%	22.1%	26.1%	24.3%	20.1%	28.7%	25.8%	28.2%	29.3%
50,000 - $74,999	25.2%	24.6%	28.1%	27.8%	16.2%	25.7%	22.1%	25.5%	21.7%	23.2%	19.1%
75,000 - $99,999	12.2%	13.3%	12.8%	16.5%	9.2%	10.5%	4.7%	13.0%	8.5%	8.2%	6.9%
100,000 - $149,999	8.5%	13.3%	9.2%	10.5%	6.3%	9.2%	8.1%	6.8%	5.3%	6.0%	3.7%
150,000 or greater	4.3%	6.1%	4.2%	5.7%	2.1%	2.6%	6.7%	3.9%	2.8%	2.2%	2.1%
**Marital Status**											
Married	0%	0%	0%	0%	0%	0%	0%	0%	0%	0%	0%
Widowed	5.9%	7.3%	8.9%	2.8%	11.3%	7.2%	11.4%	4.6%	6.3%	2.5%	3.7%
Divorced	19.8%	22.8%	24.7%	12.3%	26.1%	24.3%	29.5%	17.6%	25.2%	11.9%	17.6%
Separated	3.7%	4.2%	2.7%	4.1%	3.5%	2.6%	6.0%	3.7%	4.1%	3.1%	5.3%
Never been married	70.5%	65.7%	63.7%	80.9%	59.2%	65.8%	53.0%	74.2%	64.5%	82.4%	73.4%

*Race/ethnicity categories were not mutually exclusive (participants could select more than one).

### Measures

We administered the following measures. Two additional outcomes (loneliness and depressive symptomatology) are described in the Supplementary materials.

#### Life satisfaction

We assessed participants’ cognitive well-being with two separate measures: the Personal Wellbeing Index (PWI; The International Wellbeing Group, 2013) and the Satisfaction With Life Scale (SWLS; Diener et al., 1985). The PWI evaluates domain satisfaction across multiple areas: standard of living, health, life achievement, personal relationships, safety, community cohesion, future security, and spirituality. Because we used friendship and family satisfaction (i.e., types of personal relationship satisfaction) as predictors, we removed the item from the PWI that assesses personal relationships. Participants indicated their level of satisfaction with each of seven items on a 6-point Likert response scale from 1 (*not at all satisfied*) to 6 (*completely satisfied*). Cronbach’s alpha (α) coefficient was 0.87, indicating good internal reliability.

The SWLS is a 5-item measure that assesses global satisfaction with life. Example items include “I am satisfied with my life” and “In most ways my life is close to ideal.” Participants indicated how much they agreed with each statement on a 6-point scale from 1 (*completely disagree*) to 6 (*completely agree*). Cronbach’s α = 0.91.

Some researchers have argued that participants consider specific domains on the PWI when answering global items on the SWLS (Corrigan et al., 2013). Indeed, the PWI and SWLS scores in our dataset were strongly correlated (*r* = 0.79, *p* < 0.001). Although the two constructs are conceptually distinct, they may assess the same underlying construct. Thus, we explored combining all 13 items on both measures, as we have done in previous research (Cronbach’s α = 0.93; Kaufman et al., 2022b). Considering the correlation between the scales, our analyses use the combined responses of the two scales, which we have termed “life satisfaction.”

#### Friendship satisfaction

To examine participants’ friendship satisfaction, we used 12 items from the Friendship Network Satisfaction Scale (Kaufman et al., 2022a). Example items include “I feel close to my friends” and “I spend a lot of time socializing with my friends.” These items were rated on a 5-point scale (1 = *not at all agree* to 5 = *completely agree*). Cronbach’s α = 0.95.

#### Family satisfaction

We assessed family satisfaction with the 10-item Family Satisfaction Scale (Olson, 1982). Participants rated their level of satisfaction with items such as “the degree of closeness between family members” and “the amount of time [spent] together” from 1 (*not at all satisfied*) to 6 (*completely satisfied*). Cronbach’s α = 0.96.

#### Self-esteem

Participants were also asked to rate their level of agreement on 4 items from the Rosenberg Self-Esteem Scale (1 = *strongly disagree* to 6 = *strongly agree*; Rosenberg, 1965). Example items include “On the whole, I am satisfied with myself” and “I feel that I have a number of good qualities.” Cronbach’s α = 0.77.

#### Neuroticism

We used the International Personality Item Pool to assess neuroticism (Goldberg, 2019). Participants rated themselves on 8 items (e.g., “I get stressed out easily,” “I often feel sad”) from 1 (*not at all like me*) to 4 (*very much like me*). Cronbach’s α = 0.91.

#### Extraversion

We used the Big Five Inventory to assess extraversion (John and Srivastava, 1999). Participants were asked to rate themselves on 8 items (e.g., “talkative,” “full of energy”) from 1 (*strongly disagree*) to 5 (*strongly agree*). Cronbach’s α = 0.85.

#### Sociodemographic measures

Lastly, participants answered sociodemographic questions (age, gender, race/ethnicity, education, and income) that were used as covariates in analyses. Age was entered as a continuous variable, and gender was recoded to create a binary dummy coded variable (1 = Male; 0 = Female). Participants were dummy coded (e.g., 1 = Black; 0 = Not Black) into the following racial/ethnic groups: White, Black, Hispanic, and Other. Education was dummy coded into the following groups: Less than high school, high school graduate, some college or vocational school, college graduate, and post-graduate. Finally, income was dummy coded into the following groups: Less than $30,000; $30,000-$49,999; $50,000-$74,999; $75,000-$99,999; $100,000-$149,999; and $150,000 or greater.

## Analytic plan

We used friendship satisfaction, family satisfaction, self-esteem, neuroticism, and extraversion (our five selected variables) to predict life satisfaction (our primary outcome of interest) in the full sample, controlling for sociodemographic measures. To identify groups of people that were homogeneous (i.e., similar) to each other within each group and heterogeneous (i.e., different) from other groups, we performed latent profile analysis using Mplus (Version 8.1; Muthén and Muthén, 1998-2018). We based the latent profile analysis on continuous levels of our five predictors. We did not include life satisfaction (our primary outcome) because our goal was to see how combinations of predictors differentiate the outcome. Because our predictors were assessed on different scales (e.g., 1 to 5 vs. 1 to 6), we standardized each variable using Z-scores (*M* = 0; *SD* = 1), then ran an ascending number of latent profile analysis solutions up to eleven groups (or profiles). To evaluate the best model fit, we examined the following fit statistics: −2 Log-Likelihood (−2LL), Akaike Information Criterion (AIC), Bayesian Information Criterion (BIC), sample size-adjusted Bayesian Information Criterion (aBIC), Vuong-Lo- Mendell-Rubin Likelihood Ratio Test (VLMRT), and Lo-Mendell-Rubin Test (LMRT). A solution with lower −2LL, AIC, BIC, and aBIC represents a better fit. Notably, the VLMRT and LMRT statistically tests whether a given solution (*k*) is an improvement over a *k* – 1 solution (e.g., four vs. three profiles; Nylund et al., 2007).

## Results

### Using the five variables to predict life satisfaction

First, we added the five selected variables to a multiple regression model predicting life satisfaction, controlling for sociodemographic measures (age, gender, ethnicity, and income). All five variables significantly, and independently, predicted life satisfaction for the full sample. For singles, higher levels of friendship satisfaction (β = 0.17), family satisfaction (β = *0.25*), self-esteem (β = 0.41), extraversion (β = 0.07), and lower levels of neuroticism (β = −0.06) predicted greater happiness (all *p*s < 0.001, *R^2^* = 0.52). Because each of the five variables significantly predicted life satisfaction, this increased our confidence in choosing them as indicators to construct the latent profiles.

### Latent profiles

Using latent profile analysis, we successfully identified distinct groups of heterogeneous singles. Table 2 presents the −2LL, AIC, BIC, aBIC, VLMRT, and LMRT model fit indices for all solutions (one-group to 11-groups). The VLMRT and the LMRT determined that the 10-group solution was the optimal solution, and that 11 groups was not better as can be seen by the non-significant *p*-values. Further, all of the information criteria (AIC, BIC, aBIC) were consistent with the VLMRT and LMRT results. In addition to these emergent profiles being statistically optimal, the 10-profile solution also yielded the most interpretable groupings. Essentially, the latent profile analysis concluded there were 10 groups (or profiles) in the data that were significantly different from each other.

**TABLE 2:** Model fit indices for latent profile analyses.

Model/Solution	−2LL	AIC	BIC	aBIC	VLMRT	LMRT	Entropy
1-Profile	58498.26	58518.25	58583.45	58551.67	–	–	–
2-Profile	65453.82	65485.82	65589.56	65538.72	< 0.001	< 0.001	0.66
3-Profile	64349.38	64393.37	64536.01	64466.11	< 0.001	< 0.001	0.75
4-Profile	63708.48	63764.47	63946.02	63857.04	< 0.001	< 0.001	0.72
5-Profile	63456.76	63524.76	63745.20	63637.16	< 0.001	< 0.001	0.74
6-Profile	63156.74	63236.74	63496.09	63368.98	< 0.001	< 0.001	0.72
7-Profile	62875.70	62967.69	63265.94	63119.77	< 0.001	< 0.001	0.73
8-Profile	62624.30	62728.29	63065.44	62900.20	0.001	0.001	0.73
9-Profile	62451.38	62567.38	62943.43	62759.13	0.034	0.036	0.73
**10-Profile**	**62258.14**	**62386.14**	**62801.09**	**62597.72**	**0.010**	**0.011**	**0.75**
11-Profile	62109.68	62249.67	62703.53	62481.09	0.456	0.462	0.75

−2LL = −2 log-likelihood value; AIC = Akaike Information Criterion; BIC = Bayesian Information Criterion; aBIC = Adjusted Bayesian Information Criterion; VLMRT = Vuong-Lo-Mendell-Rubin Likelihood Ratio Test; LRMT = Lo-Mendell-Rubin Test. Bold values represent the best fitting model/solution.

**FIGURE 1:**
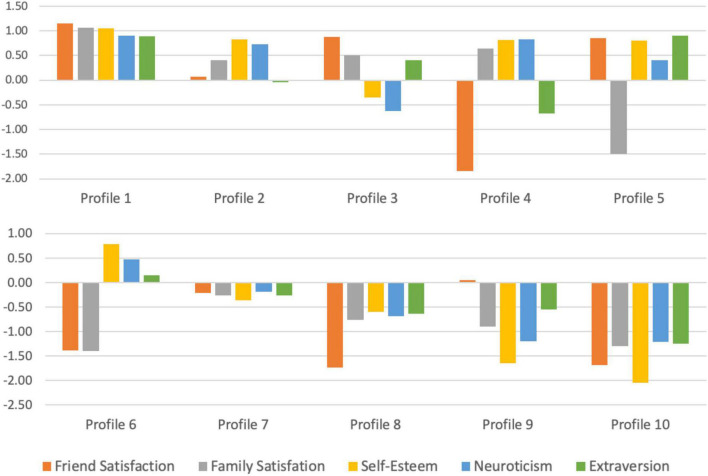
Relationship, self-esteem, and personality patterns for each profile. Standardized means for predictor variables used in latent profile analysis by profile, ordered from most happy profile (Profile 1) to least happy profile (Profile 10). For ease of interpretation, neuroticism is reversed so positive means indicate lower levels and negative means indicate higher levels.

To ease interpretation, we ordered the 10 profiles based on our primary outcome (life satisfaction) from most happy (Profile 1) to least happy (Profile 10). We discuss how the 10 profiles map onto life satisfaction in greater detail below. First, we describe how each profile is descriptively and conceptually different in terms of the five predictors and demographics (see Tables 1, 3, and 4, as well as Figure 1).

**TABLE 3:** Standardized descriptive statistics by profile.

		Life satisfaction	Friend satisfaction	Family satisfaction	Self-esteem	Neuroticism	Extraversion
		
Profile	*n (%)*	*M (SD)*	*M (SD)*	*M (SD)*	*M (SD)*	*M (SD)*	*M (SD)*
Profile 1	670 (13.9%)	1.02 (0.69)	1.15 (0.35)	1.06 (0.53)	1.05 (0.48)	−0.90 (0.76)	0.89 (0.81)
Profile 2	912 (18.9%)	0.40 (0.68)	0.07 (0.46)	0.41 (0.63)	0.83 (0.45)	−0.73 (0.69)	−0.01 (0.82)
Profile 3	716 (14.8%)	0.28 (0.81)	0.88 (0.41)	0.50 (0.65)	−0.35 (0.51)	0.62 (0.74)	0.41 (0.80)
Profile 4	142 (2.9%)	0.22 (0.89)	−1.84 (0.39)	0.64 (0.69)	0.81 (0.53)	−0.83 (0.78)	−0.68 (0.95)
Profile 5	152 (3.1%)	0.22 (0.79)	0.85 (0.53)	−1.49 (0.53)	0.80 (0.53)	−0.41 (0.74)	0.90 (0.83)
Profile 6	149 (3.1%)	−0.19 (0.98)	−1.39 (0.52)	−1.40 (0.61)	0.79 (0.46)	−0.48 (0.81)	0.15 (0.94)
Profile 7	1269 (26.2%)	−0.31 (0.71)	−0.21 (0.44)	−0.26 (0.69)	−0.36 (0.48)	0.19 (0.70)	−0.26 (0.78)
Profile 8	318 (6.6%)	−0.82 (0.86)	−1.74 (0.43)	−0.76 (0.88)	−0.60 (0.54)	0.69 (0.72)	−0.63 (0.96)
Profile 9	319 (6.6%)	−1.04 (0.84)	0.05 (0.59)	−0.90 (0.86)	−1.65 (0.50)	1.20 (0.58)	−0.55 (1.02)
Profile 10	188 (3.9%)	−1.62 (0.67)	−1.69 (0.50)	−1.30 (0.84)	−2.05 (0.46)	1.21 (0.63)	−1.25 (0.83)

Standardized using *Z*-scores (full sample *M* = 0; *SD* = 1).

**TABLE 4:** Unstandardized life satisfaction for the full sample and each profile.

Sample/Profile	*M*	*SD*	%
Full Sample	3.85	1.09	64.2%
Profile 1	4.97	0.76	82.8%
Profile 2	4.29	0.74	71.5%
Profile 3	4.15	0.89	69.2%
Profile 4	4.09	0.98	68.2%
Profile 5	4.09	0.86	68.2%
Profile 6	3.65	1.07	60.8%
Profile 7	3.52	0.78	58.7%
Profile 8	2.96	0.94	49.3%
Profile 9	2.72	0.92	45.3%
Profile 10	2.07	0.73	34.5%

Life satisfaction scores ranged from 1 (minimum) to 6 (maximum). “%” indicates what percent of the maximum possible life satisfaction score the mean of the full sample and each profile represents.

#### Profile 1: Best relationships, self-esteem, and personality

Examining standardized means, participants in Profile 1 (*n* = 670; 13.9% of the sample) had optimal levels of all five predictors. They had the best relationships (very high friend [*M* = 1.15] and family [*M* = 1.06] satisfaction), the best self-esteem (*M* = 1.05), and the best personality traits (low neuroticism [*M* = −0.90] and high extraversion [*M* = 0.89]). Demographically, Profile 1 had the highest mean income (unstandardized *M* = 2.99), as well as the highest percentage of those earning $100,000 to $149,999 (13.3%) and the lowest percentage of those earning less than $30,000. This group also had the highest percentage of singles who had earned post-graduate degrees (0.4%).

#### Profile 2: Good relationships, self-esteem, and personality

Profile 2 (*n* = 912; 18.9%) had good relationships (average friend [*M* = 0.07] and somewhat high family [*M* = 0.41] satisfaction), high self-esteem (*M* = 0.83), and good (but not great) personality traits (low neuroticism [*M* = −0.73] and average extraversion [*M* = −0.01]). Demographically, Profile 2 had the highest levels of mean education (unstandardized *M* = 3.59), as well as the highest percentage of college graduates (18.2%) and the lowest percentage of individuals with less than a high school degree (15.0%). Profile 2 also had the highest percentage of those earning $50,000 to $74,999 (28.1%).

#### Profile 3: Very good relationships, somewhat low self-esteem, and mixed personality

Profile 3 (*n* = 716; 14.8%) had very good relationships (high friend [*M* = 0.88] and family [*M* = 0.50] satisfaction), somewhat low self-esteem (*M* = −0.35), and mixed personality traits (high neuroticism [*M* = 0.62] and extraversion [*M* = 0.41]). Overall, participants in this group were relatively well off except for their somewhat low self-esteem and high neuroticism. Demographically, Profile 3 was one of the youngest (*M*_*age*_ = 33.00), least White (65.1%) and most Hispanic (18.0%) groups.

#### Profile 4: Mixed relationships, good self-esteem, and mixed personality

The smallest group, Profile 4 (*n* = 142; 2.9%), had mixed relationships (very low friend [*M* = −1.84] and high family [*M* = 0.64] satisfaction), high self-esteem (*M* = 0.81), and mixed personality traits (low neuroticism [*M* = −0.83] and extraversion [*M* = −0.68]). Notably, what stood out most about this relatively adaptive group was their low friendship satisfaction and extraversion. Demographically, participants in Profile 4 were the oldest (*M*_*age*_ = 50.6), most male (48.6%), least Hispanic (6.3%), and had the largest percentage of those coming from “Other” ethnicities (7.0%; e.g., Native American, Pacific Islander). Profile 4 also had the highest percentage of people earning less than $30,000 per year (40.1%).

#### Profile 5: Mixed relationships, good self-esteem, and good personality

Profile 5 (*n* = 152; 3.1%) had mixed relationships (high friend [*M* = 0.85] and very low family [*M* = −1.49] satisfaction), high self-esteem (*M* = 0.80), and good personality traits (somewhat low neuroticism [*M* = -0.41] and high extraversion [*M* = 0.90]). Strikingly, this otherwise well-off group had very low family satisfaction. Demographically, Profile 5 had the largest percentage of women (70.4%) and the lowest percentage of separated people (6.0%).

#### Profile 6: Very bad relationships, good self-esteem, and good personality

Profile 6 (*n* = 149; 3.1%) had very bad relationships (very low friend [*M* = −1.39] and family [*M* = −1.40] satisfaction), high self-esteem (*M* = 0.79), and good personality traits (somewhat low neuroticism [*M* = −0.48] and average extraversion [*M* = 0.15]). It is noteworthy that next to Profile 10 (the worst-off group), Profile 6 had the worst friend and family relationships. Demographically, participants in Profile 6 were some of the oldest (*M*_*age*_ = 50.1), most Black (18.8%), and least Asian (3.4%). They also had the lowest percentage of high school graduates (26.8%) and the highest percentage of people with at least some college or vocational school (38.9%). In terms of income, Profile 6 had the lowest percentages of those earning $30,000 to $49,999 (20.1%) and $75,000 to $99,999 (4.7%), as well as the highest percentage of those earning over $150,000 (6.7%). Finally, they also had the highest percentages of people who were widowed (11.4%), divorced (29.5%), and separated (6.0%), as well as the lowest percentages of people who had never been married (53.0%).

#### Profile 7: Just below average relationships, self-esteem, and personality

The largest group, Profile 7 (*n* = 1269; 26.2% of the sample), had just below average relationships (somewhat low friend [*M* = −0.21] and family [*M* = −0.26] satisfaction), self-esteem (*M* = −0.36), and personality traits (somewhat high neuroticism [*M* = 0.19] and somewhat low extraversion [*M* = −0.26]). Demographically, Profile 7 had one of the highest percentages of men (46.9%) and Asians (8.8%).

#### Profile 8: Bad relationships, self-esteem, and personality

Profile 8 (*n* = 318; 6.6%) had bad relationships (very low friend [*M* = −1.74] and low family [*M* = −0.76] satisfaction), low self-esteem (*M* = −0.60), and problematic personality traits (high neuroticism [*M* = 0.69] and low extraversion [*M* = −0.63]). Although, all five predictor variables were trending in an undesirable direction, Profile 8’s very low friendship satisfaction stood out. There was nothing particularly unique about Profile 8’s demographic make-up.

#### Profile 9: Mixed relationships, very bad self-esteem, and bad personality

Profile 9 (*n* = 319; 6.6%) had mixed relationships (average friend [*M* = 0.05] and low family [*M* = −0.90] satisfaction), very low self-esteem (*M* = −1.65), and bad personality traits (very high neuroticism [*M* = 1.20] and low extraversion [*M* = −0.55]). Demographically, Profile 9 was the youngest (*M*_*age*_ = 32.73) and highly female (68.3%). They also had the highest percentage of high school graduates (34.5%) and the lowest percentage of college graduates (4.7%). Finally, participants in Profile 8 had the lowest percentages of people who were widowed (2.5%) and divorced (11.9%), as well as the highest percentage of people who had never been married (82.4%).

#### Profile 10: Worst relationships, self-esteem, and personality

Finally, Profile 10 (*n* = 188; 3.9% of the sample) had the worst relationships (very low friend [*M* = −1.69] and family [*M* = -1.30] satisfaction), the worst self-esteem (*M* = −2.05), and the worst personality traits (very high neuroticism [*M* = 1.21] and very low extraversion [*M* = −1.25]).

Demographically, Profile 10 had the largest percentage of White people (80.9%) and the lowest percentage of Black people (9.6%). They were the least educated (*M* = 2.95), with the highest percentage of people with less than a high school diploma (31.9%) and the smallest percentage of people with some college (27.7%). Profile 10 also had the lowest levels of income (*M* = 2.14), including the smallest percentages of those earning $100,000 to $149,999 (3.7%) and over 150,000 (2.1%).

#### How do profiles map onto life satisfaction?

Notably, the full sample’s life satisfaction mean (before standardization) was 3.85, which falls between response options 3 and 4 on the 6-point scale. This suggested that, on average, singles were somewhat satisfied with their lives. When examining each profiles’ mean life satisfaction, the 10 profiles could be broadly categorized into *happy profiles* (Profiles 1-5 with life satisfaction above the full sample’s mean) and *unhappy profiles* (Profiles 6-10 with life satisfaction scores below the mean; see Table 3).

##### Happy profiles

Roughly half of our single participants (those in 5 of the 10 profiles) were relatively happy (Profiles 1-5; 53.6% of the sample). These profiles’ life satisfaction *Z*-scores ranged from + 1.02 (Profile 1) to + 0.22 (Profile 5). Further, Profiles 1 (unstandardized *M* = 4.97) and 2 (unstandardized *M* = 4.29), which constituted 32.8% of the sample, were near the top of the 6-point scale, with their means representing 82.8 and 71.5% of the maximum possible score, respectively (see Table 3). These percentages are relatively consistent with thresholds set forth by Diener (71.4% and above) as people who are highly satisfied with their lives; for such people “life is enjoyable” and “the major domains of life are going well” (e.g., work/school, family, leisure, personal development; Diener et al., 1985; Diener, 2006).

##### Unhappy profiles

The other half of our single participants were relatively unhappy (Profiles 6-10; 46.4% of the sample). These profiles’ life satisfaction *Z*-scores ranged from −0.19 (Profile 6) to −1.62 (Profile 10). However, even Profile 10 (the unhappiest profile) still had an unstandardized mean life satisfaction (*M* = 2.21) that was at 34.5% of the maximum possible level of thresholds set forth by Diener (see Table 3). On average, even Profile 10 did not report that they were “1 = *not at all satisfied*” with their lives.

##### Happy vs. unhappy profiles

It is noteworthy that profiles with more desirable personal relationship, self-esteem, and personality patterns (i.e., high friend satisfaction, family satisfaction, self-esteem, and extraversion, as well as low neuroticism) had higher levels of life satisfaction; profiles with less desirable patterns had lower life satisfaction. For example, Profile 1 had the best relationships, self-esteem, and personality traits, as well as the highest life satisfaction. Conversely, Profile 10 had the worst relationships, self-esteem, and personality traits, as well as the lowest life satisfaction. However, disadvantages on one or two predictor variables were often offset by advantages on others. For example, happy Profile 4 had low friend satisfaction and extraversion, but this was offset by their high family satisfaction, high self-esteem, and low neuroticism.

### Additional/supplemental analyses

We conducted additional analyses reported in the online Supplementary materials that included: (1) examining whether associations varied by profile (see Supplementary Table 1); (2) exploring differences by dating status (i.e., daters vs. non-daters; see Supplementary Tables 2–6); (3) further validating the profiles with other well-being-related outcomes (loneliness and depressive symptomatology; see Supplementary Tables 7–8); and (4) summarizing bivariate correlations (see Supplementary Table 9).

## Discussion

Overall, our study provides additional insight into the lives of singles, a group of people who have been historically understudied. Specifically, we wanted to examine singles’ happiness, so we focused on five variables that are among the strongest predictors of well-being (friendship satisfaction, family satisfaction, self-esteem, neuroticism, and extraversion). Along the way, we addressed a few previously unanswered research questions.

First, how well do our five selected variables predict life satisfaction? In multiple regression models, all five variables significantly and uniquely predicted life satisfaction in the full sample of singles. Singles who were more satisfied with themselves, their friends, and their family were relatively happier. Those who were more extraverted and less neurotic were also happier, but personality had a smaller impact on well-being.

Second, could we identify groups (or profiles) to better differentiate singles? By entering our five predictors into latent profile analysis, we successfully identified 10 profiles that better portrayed the complex landscape of American singles. Notably, patterns among the five predictors that captured aspects of singles’ personal relationships, self-esteem, and personality mapped onto varying levels of singles’ happiness. When ordered based on life satisfaction, the happiest singles (Profiles 1-2) had good to very good personal relationships (high friend and family satisfaction), self-esteem, and personality traits (high extraversion and low neuroticism). The least happy singles (Profile 10) were the worst off in terms of their personal relationships (low friend and family satisfaction), self-esteem, and personality traits (low extraversion and high neuroticism). In between these extremes (especially in the moderately happy groups), we found interesting nuances. Namely, negative patterns in one or two predictors were often offset by positive patterns in others. For example, Profile 3 (a happy group) had somewhat low self-esteem and high neuroticism but made up for this with high friendship satisfaction, family satisfaction, and extraversion. Similarly, Profile 4 (another happy group) had low friendship satisfaction and extraversion but made up for this with high family satisfaction and self-esteem and low neuroticism.

We also found it useful to examine the demographic composition of each profile to learn more about the singles in each group. For example, the happiest profile (1) had high levels of education and income, while the unhappiest profile (10) had the lowest levels. This may make sense given that life satisfaction is positively associated with income and education (Meeks and Murrell, 2001; Killingsworth, 2021). Additionally, the oldest singles in Profile 6 (who were more likely to be widowed, divorced, or separated) had very low friend and family satisfaction. Notably, adults tend to have fewer friendships as they age (Bhattacharya et al., 2016), but older adults often benefit socially and emotionally from interacting with their friends (Larson et al., 1986; Lee and Ishii-Kuntz, 1987; Huxhold et al., 2013). Being widowed, having a bad previous marriage, and/or raising children with a contentious ex-spouse may partly explain Profile 6’s higher levels of family dissatisfaction. In contrast, the youngest singles in Profile 9 (a very unhappy group) had average friendship satisfaction, but low family satisfaction, self-esteem, and extraversion, as well as high neuroticism. These findings may be partially explained by research showing that younger people (especially those belonging Generation Z) have dramatically decreased self-esteem and life satisfaction, relative to previous, older generations (Twenge et al., 2018).

### Strengths, limitations, and future directions

This study has several strengths, which lend further confidence to our findings. First, we collected a large, high-powered sample, and such samples tend to yield more accurate and stable effect size estimates (Funder and Ozer, 2019). To our knowledge, ours is one of the largest studies on unpartnered singles to date, with only a few studies with similar *N*s (Brown, 2020; Park et al., 2022). Second, we matched our sample to United States census targets, making it diverse in terms of age, ethnicity, education, and income. This allowed us to determine whether our results generalized across different demographic groups, as well as examine how specific groups of singles differ from each other demographically. Third, we used well-cited, expansive, and reliable measures of life satisfaction. Finally, we primarily focused on person-centered analyses to examine singles’ well-being.

Nevertheless, our study is also subject to several limitations that may inform future research. First, because our data were collected in the United States, an oversampled “WEIRD” (Western, Educated, Industrialized, Rich, and Democratic) country, our results may not generalize to other nations, cultures, and contexts (Henrich et al., 2010). Future research could explore how the associations reported here vary by culture. Second, ours was an exploratory study without *a priori* hypotheses, so future researchers should replicate these findings in preregistered studies. Third, because this was a cross-sectional study, we cannot infer causality. For example, we cannot definitively state that friendship and family satisfaction *cause* superior well-being. It could be that a third unmeasured variable is driving higher levels of friendship satisfaction, family satisfaction, and well-being. Future studies could employ positive activity interventions aimed at improving relationship satisfaction to determine its effects on well-being. Fifth, different researchers could select different variables that predict well-being to form profiles (e.g., meaning in life, self-compassion, autonomy), which may alter results. Relatedly, satisfaction with singlehood (e.g., how happy singles are with being single, their sexual/intimate relations, etc.), as well as other personality traits like openness, conscientiousness, and agreeableness may be other important predictors of relationship satisfaction and life satisfaction that researchers could explore in future studies. Further, because our study used two separate personality measures to assess extraversion (IPIP) and neuroticism (BFI), it would be useful to see whether these results replicate when all personality traits are assessed with the same measure (e.g., the BFI-2). Additionally, a limitation of the present study is that it does not apply both taxometric and latent profile analyses to compare dimensional vs. categorical latent variable structures. A separate study of singles using taxometric analysis could be a useful approach in future research. Finally, we did not compare groups of singles to groups of spouses. Even though most singles were fairly to very happy, we cannot conclude that some singles are happier than some spouses. Future studies should directly compare profiles of single vs. coupled people to better address this issue.

## Conclusion

Most people want to be happy (Diener and Seligman, 2002), and our research suggests that (contrary to popular thought) most single, unpartnered people are fairly to very satisfied with their lives. Most of all, our findings suggest that single adults who have positive relationships—with both themselves and others—are happiest. However, satisfying relationships with both friends and family are not always required for singles to be happy; sometimes having just good friends or just good family will do (especially if other positive elements are present, like high self-esteem and/or extraversion). Finally, researchers who attempt to distill singles down to one mean value potentially obfuscate more nuanced groups (or types) of single people revealed by latent profile analysis. Overall, this knowledge is vital given that the share of unpartnered single people continues to increase in both the United States and abroad. Clinicians should be aware of our findings and be prepared to probe the strengths and weaknesses of singles’ personal relationships, self-esteem, and personality. They may also want to customize their approach to the type of single person they are treating (i.e., by understanding the attributes of the profile into which that person likely falls). Finally, future interventions aimed at improving personal relationships and self-esteem could be used by both researchers and practitioners to advance the well-being of singles.

## Data availability statement

The raw data supporting the conclusions of this article will be made available by the authors, without undue reservation.

## Ethics statement

The studies involving human participants were reviewed and approved by UCLA Office of the Human Research Protection Program (OHRPP). The patients/participants provided their written informed consent to participate in this study.

## Author contributions

VK, AG, and LS oversaw survey design, data collection, and data cleaning. VK, AR, and LW conducted analyses and created/formatted tables. LW and VK co-wrote the manuscript. All authors contributed to manuscript revision, read, and approved the submitted version.
