# Supplementary material for: Expanding relationship science to unpartnered singles: What predicts life satisfaction?
Source: Front Psychol. 2022 Sep 16;13:904848. doi: 10.3389/fpsyg.2022.904848 (PMC9523881; doi:10.3389/fpsyg.2022.904848)
Supplement: Supplementary file 1 [file Data_Sheet_1.docx]

**SUPPLEMETAL MATERIALS**

*Expanding Relationship Science to Unpartnered Singles: What Predicts Life Satisfaction?*

Lisa C. Walsh, Ariana M. Gonzales, Lucy Shen, Anthony Rodriguez, and Victor A. Kaufman

**Do Associations Vary by Profile?**

We ran multiple regression models predicting life satisfaction from the five predictor variables to examine associations within each profile (see Table S1). Three variables emerged as strong predictors in most profile-specific models: self-esteem (9 significant βs ranging from .29 to .91, all *p*s<.001), family satisfaction (7 significant βs ranging from .13 to .37, most *p*s<.001), and friendship satisfaction (6 significant βs ranging from .09 to .28, *p*s=.038 to <.001). Self-esteem was usually the strongest predictor in each model. However, patterns differed by profile. For example, in Profiles 1 and 3, family satisfaction (β=.34 and .37, respectively) was a stronger predictor than self-esteem (β=.29 and .31). Additionally, neuroticism (3 significant βs ranging from -.11 to -.16, all *p*s<.001) and extraversion (4 significant βs ranging from .09 to .18, *p*s=.028 to .001) frequently become non-significant predictors. Essentially, neuroticism and extraversion tended to fall away and be subsumed by the other three variables in the models, with respect to unique variances.

**Table S1***Profile-Specific Regression Models*

|  |  | Friend  Satisfaction | | Family  Satisfaction | | Self-Esteem | | Neuroticism | | Extraversion | |
| --- | --- | --- | --- | --- | --- | --- | --- | --- | --- | --- | --- |
|  | *R^2^* | *β* | *p* | *β* | *p* | *β* | *p* | *β* | *p* | *β* | *p* |
| Profile 1 | .20 | **.23** | .001 | **.34** | <.001 | **.29** | <.001 | **-.16** | <.001 | **.09** | .005 |
| Profile 2 | .18 | **.09** | .038 | **.18** | <.001 | **.46** | <.001 | **-.11** | <.001 | .00 | .942 |
| Profile 3 | .24 | **.17** | .013 | **.37** | <.001 | **.31** | <.001 | .02 | .612 | **.12** | .001 |
| Profile 4 | .13 | .31 | .126 | .00 | .982 | .23 | .139 | -.03 | .810 | -.01 | .86 |
| Profile 5 | .28 | **.28** | .027 | -.04 | .743 | **.47** | <.001 | -.13 | .130 | -.08 | .287 |
| Profile 6 | .32 | .01 | .924 | .11 | .384 | **.91** | <.001 | -.11 | .230 | **.18** | .028 |
| Profile 7 | .23 | **.22** | <.001 | **.28** | <.001 | **.44** | <.001 | **-.11** | <.001 | **.07** | .004 |
| Profile 8 | .22 | .01 | .923 | **.24** | <.001 | **.52** | <.001 | .02 | .758 | .07 | .15 |
| Profile 9 | .21 | **.21** | .007 | **.23** | <.001 | **.48** | <.001 | -.08 | .337 | .06 | .168 |
| Profile 10 | .20 | .08 | .403 | **.13** | .028 | **.42** | <.001 | .00 | .96 | -.03 | .586 |

*Note.* Dependent Variable: Life Satisfaction. All regression models controlled for sociodemographic measures (age, gender, ethnicity, education, and income). **Bold** beta estimates are significant at *p*<.05.

**Daters vs. Non-daters**

Singles may also differ in terms of their interest in dating (or lack thereof). Seeking a long-term romantic partner may partially act as a substitute for having one—or, alternatively, make its lack more salient. According to recent Pew Research Center data, half (50%) of singles are looking for a committed romantic relationship or casual dates (i.e., they are daters), while the other half are not (i.e., they are non-daters; Brown, 2020). Dating-driven differences may also be related to other individual differences, such as personality and friendship and family satisfaction.

Relevant to these considerations are the distinctive attributes of daters vs. non-daters, who have variable demographics and experiences. Demographically, women are less likely to date than men, and older people (65+) are less likely to date than younger people (ages 18-64). Experientially, many daters report that their dating lives are not going very well, but others are fairly to very happy with their dating experiences (Brown, 2020). Dating also comes with challenges, especially for women, who are often the targets of threats, stalking, and other forms of sexual harassment. On the other side of the dating divide, non-daters frequently report feeling satisfied with their singlehood. Many non-daters say they have more important priorities or “just like being single.”

Daters may also differ psychologically from non-daters, and this may go in different directions for different reasons. Indeed, daters may be more (or less) happy than non-daters. Perhaps daters are happier than non-daters because dating involves conversations with strangers, which can improve well-being (Gunaydin et al., 2021; Schroeder et al., 2021). There may also be self-selection effects. Happy people are more likely to get married than their less happy peers, so it may also be the case that happy people are more likely to date (Stutzer & Frey, 2006). Alternatively, daters may be less happy than non-daters due to difficulty in finding dates or being harassed (Brown, 2020). However, if dating experiences do not alter well-being much, daters and non-daters may be equally happy. Further, differences in happiness may, in turn, be linked to differences in other variables. Given the sociable nature of dating, daters may be more extraverted than non-daters. It may also be the case that non-daters are unmotivated to date because they are more satisfied with their existing friend and/or family relationships.

**Dating Status**

To determine singles’ interest in dating (or lack thereof), we asked them to “select the choice(s) that best describe your interest in dating before the pandemic (select all that apply).” They were given three response options that included: (1) “Interested in casually dating, without the intention of entering a committed romantic relationship,” (2) “Interested in dating with the intention of committed romantic relationship,” and (3) “Not interested in dating at all.” Using this question, participants who selected options 1 and/or 2 were classified as daters, while those who selected option 3 were classified as non-daters.

**Do Associations Vary by Dating Status?**

We examined differences by dating subgroups, comparing daters (70.1% of the sample) to non-daters (29.9% of the sample). See Table S2 for demographics and Table S3 for standardized descriptive statistics for daters and non-daters. In multiple regression models, the associations did not differ much by dating status (see Table S4). All five variables significantly predicted life satisfaction for daters and non-daters (all *p*s<.001, *R^2^*=.52 to .54)—with the exception that extraversion did not predict life satisfaction for non-daters (β*=.02, p*=.349). For daters and non-daters, higher levels of friendship satisfaction (daters β*=*.18; non-daters β*=*.15), family satisfaction (β=.25; .22), self-esteem (β=.37; .49), extraversion (β=.10; .02), and lower levels of neuroticism (β=-.04; -.11) predicted greater happiness.

**Do Daters and Non-Daters Differ from Each Other?**

To address this question, we conducted independent samples *t*-tests on the full sample. (see Table S5). Daters’ (*M*=3.85) and non-daters’ (*M*=3.85) unstandardized life satisfaction means were identical. Daters were not more satisfied with their lives than non-daters (*t*[4833]=.21, *p*=.837, *d*=.01), nor did they have higher family satisfaction (*t*[4833]=.65, *p*=.52, *d*=.02). However, daters had significantly higher levels of friendship satisfaction (*t*[4833]=10.94, *p* <.001, *d*=.34), neuroticism (*t*[4833]=6.59, *p*<.001, *d*=.21), and extraversion (*t*[4833]=10.14, *p*<.001, *d*=.32) than non-daters, as well as lower self-esteem (*t*[4833]=-6.87, *p*<.001, *d*=-0.22). Demographically, daters were more likely to be younger (*M*_age_=38), male (52.8% female), ethnically diverse (68.5% White), earn higher income, and never be married (74.4%), relative to non-daters (*M*_age_=47.5; 68.5% female; 77.3% White; 61.5% never married).

**Do Daters and Non-daters Differ Within Each Profile?**

To answer this question, we examined means and independent samples *t*-tests within each profile (see Tables S5 and S6). Due to multiple comparisons, we Bonferroni corrected our alpha level to *p*<.0007. At this threshold, Profile 2 showed a significant difference in life satisfaction between daters and non-daters (*t*[910]=-4.03, *p*<.0001, *d*=-.28), such that daters were less satisfied with their lives than non-daters. In Profile 2, daters also had higher friend satisfaction (*t*[910]=4.30, *p*<.0001, *d*=.30), lower family satisfaction (*t*[910]=-3.61, p=.0003, *d*=-.25), lower self-esteem (*t*[910]=-5.81, *p*<.0001, *d*=-.35), and higher extraversion (*t*[910]=3.75, p=.0002, *d*=.26) than non-daters. However, in Profile 3, daters were happier than non-daters (*t*[714]=3.57, p=.000, *d*=.36). The only other profile dating differences were seen in: Profile 1, where daters had lower self-esteem than non-daters (*t*[668]=-5.19, p<.0001, *d*=-.46); and in Profile 7, where daters were more extraverted than non-daters (*t*[1267]=5.36, p<.0001, *d*=.33). Overall, across the other profiles, daters tended to have relatively similar levels of life satisfaction as non-daters, as well as similar levels of friend satisfaction, family satisfaction, self-esteem, neuroticism, and extraversion.

**Table S2**

*Demographics of Daters and Non-daters*

| Characteristic | Daters  (*n*=3389; 70.1%) | Non-daters  (n=1,446; 29.9%) |
| --- | --- | --- |
| Age (*M* ± *SD* in years)­ | 38.0 (14.6) | 47.5 (14.7) |
| Sex (% Female) | 52.8% | 68.5% |
| Race/Ethnicity* |  |  |
| White/Caucasian | 68.5% | 77.3% |
| Black/African American | 15.8% | 11.4% |
| Hispanic/Latino(a) | 15.5% | 9.7% |
| Asian | 7.4% | 4.7% |
| Other | 2.8% | 3.0% |
| Education Level |  |  |
| Less than high school | 1.7% | 2.5% |
| High school graduate | 19.1% | 22.7% |
| Some college/vocational | 29.3% | 30.2% |
| College graduate | 36.7% | 31.7% |
| Post-graduate | 13.2% | 12.7% |
| Prefer not to answer | 0.1% | 0.3% |
| Household Income |  |  |
| Less than $30,000 | 19.7% | 32.1% |
| $30,000 - $49,999 | 26.3% | 26.4% |
| $50,000 - $74,999 | 25.7% | 23.9% |
| $75,000 - $99,999 | 13.7% | 8.8% |
| $100,000 - $149,999 | 9.4% | 6.4% |
| $150,000 or greater | 5.0% | 2.4% |
| Marital Status |  |  |
| Married | 0% | 0% |
| Widowed | 3.9% | 10.7% |
| Divorced | 17.8% | 24.6% |
| Separated | 3.9% | 3.3% |
| Never been married | 74.4% | 61.5% |

*Note.* *Race/ethnicity categories were not mutually exclusive (participants could select more than one).

**Table S3**

*Standardized Descriptive Statistics for Daters and Non-daters*

|  | Life Satisfaction | Friend Satisfaction | Family Satisfaction | Self-Esteem | Neuroticism | Extraversion |
| --- | --- | --- | --- | --- | --- | --- |
| Subgroup | *M (SD)* | *M (SD)* | *M (SD)* | *M (SD)* | *M (SD)* | *M (SD)* |
| Daters | .00 (.97) | .10 (.96) | .01 (.97) | -.06 (.97) | .06 (1.00) | .09 (.97) |
| Non-daters | .00 (.96) | -.24 (1.06) | -.01 (1.06) | .15 (1.06) | -.14 (.99) | -.22 (1.03) |

*Note.* Standardized using Z-scores (full sample *M*=0; *SD=*1).

**Table S4**

*Regression Models for Daters and Non-daters*

|  |  | Friend  Satisfaction | | Family  Satisfaction | | Self-Esteem | | Neuroticism | | Extraversion | |
| --- | --- | --- | --- | --- | --- | --- | --- | --- | --- | --- | --- |
|  | *R^2^* | *β* | *p* | *β* | *p* | *β* | *p* | *β* | *p* | *β* | *p* |
| Daters | .52 | **.18** | <.001 | **.25** | <.001 | **.37** | <.001 | **-.04** | .008 | **.10** | <.001 |
| Non-Daters | .54 | **.15** | <.001 | **.22** | <.001 | **.49** | <.001 | **-.11** | <.001 | .02 | .349 |

*Note.* Dependent Variable: Life Satisfaction. All regression models controlled for sociodemographic measures (age, gender, ethnicity, education, and income). **Bold** beta estimates are significant at *p*<.05.

**Table S5**

*Independent Samples t-tests Comparing Daters vs. Non-daters in the Full Sample and Each Profile*

|  | Life  Satisfaction | | Friend  Satisfaction | | Family  Satisfaction | | Self-Esteem | | Neuroticism | | Extraversion | |
| --- | --- | --- | --- | --- | --- | --- | --- | --- | --- | --- | --- | --- |
|  | *t* | *p* | *t* | *p* | *t* | *p* | *t* | *p* | *t* | *p* | *t* | *p* |
| Full Sample | 0.21 | .837 | **10.94** | **<.001** | 0.65 | .517 | **-6.87** | **<.001** | **6.59** | **<.001** | **10.14** | **<.001** |
| Profile 1 | -2.07 | .039 | 2.00 | .046 | -1.11 | .269 | **-5.19** | **<.001** | 0.92 | .359 | 2.78 | .006 |
| Profile 2 | **-4.03** | **<.001** | **4.30** | **<.001** | **-3.61** | **<.001** | **-5.06** | **<.001** | 0.88 | .381 | **3.75** | **<.001** |
| Profile 3 | **3.57** | **<.001** | -2.12 | .0347 | 0.64 | .521 | -0.93 | .351 | 0.63 | .529 | 3.21 | .001 |
| Profile 4 | -1.93 | .056 | 1.74 | .085 | -0.87 | .385 | -0.44 | .657 | 0.45 | .652 | 1.12 | .264 |
| Profile 5 | 1.17 | .246 | -0.33 | .743 | 2.54 | .012 | 0.32 | .752 | 1.44 | .151 | 1.83 | .069 |
| Profile 6 | -0.31 | .757 | -0.62 | .538 | -1.38 | .168 | -0.25 | .805 | 1.61 | .111 | 1.57 | .112 |
| Profile 7 | -0.02 | .986 | 2.75 | .006 | 0.72 | .471 | -3.17 | .002 | 0.48 | .632 | **5.36** | **<.001** |
| Profile 8 | 1.24 | .217 | 2.01 | .045 | -1.17 | .242 | 0.04 | .968 | 2.02 | .044 | 2.32 | .021 |
| Profile 9 | 1.74 | .083 | 1.85 | .065 | -0.72 | .470 | 2.29 | .023 | 1.58 | .115 | 2.53 | .012 |
| Profile 10 | -1.15 | .250 | 0.84 | .401 | -0.78 | .438 | -1.08 | .280 | 1.87 | .062 | 0.63 | .533 |

*Note.* **Bold** indicates there was a statistically significant difference between Daters and Non-daters using 2-tailed independent samples *t*-tests that were Bonferroni-corrected (p < .0007). Positive *t* estimates indicate that Dater means are higher than Non-dater means. Negative *t* estimates indicate that Dater means are lower than Non-dater means.

**Table S6**

*Standardized Descriptive Statistics for Daters and Non-daters Within Each Profile*

|  |  | Life  Satisfaction | Friend  Satisfaction | Family  Satisfaction | Self-Esteem | Neuroticism | Extraversion |
| --- | --- | --- | --- | --- | --- | --- | --- |
|  | *n (%)* | *M (SD)* | *M (SD)* | *M (SD)* | *M (SD)* | *M (SD)* | *M (SD)* |
| Profile 1 Daters | 493 (73.6%) | .99 (.69) | 1.16 (.34) | 1.04 (.52) | **1.00 (.49)** | -.89 (.77) | .95 (.80) |
| Profile 1 Non-daters | 177 (26.4%) | 1.11 (.71) | 1.10 (.38) | 1.10 (.56) | **1.21 (.42)** | -.95 (.74) | .75 (.82) |
| Profile 2 Daters | 572 (62.7%) | **.33 (.65)** | **.12 (.45)** | **.35 (.59)** | **.77 (.45)** | -.71 (.69) | **.07 (.79)** |
| Profile 2 Non-daters | 340 (37.3%) | **.52 (.71)** | **-.01 (.48)** | **.50 (.67)** | **.93 (.44)** | -.75 (.69) | **-.14 (.86)** |
| Profile 3 Daters | 599 (83.7%) | **.32 (.79)** | .86 (.41) | .51 (.64) | -.36 (.49) | .63 (.74) | .45 (.77) |
| Profile 3 Non-daters | 117 (16.3%) | **.03 (.88)** | .95 (.41) | .47 (.71) | -.31 (.59) | .58 (.71) | .19 (.94) |
| Profile 4 Daters | 54 (38.0%) | .03 (.82) | -1.76 (.39) | .58 (.63) | .78 (.51) | -.79 (.73) | -.57 (.93) |
| Profile 4 Non-daters | 88 (62.0%) | .33 (.92) | -1.88 (.39) | .68 (.72) | .82 (.54) | -.85 (.82) | -.75 (.96) |
| Profile 5 Daters | 106 (69.7%) | .27 (.77) | .84 (.53) | -1.42 (.53) | .81 (.48) | -.35 (.72) | .98 (.81) |
| Profile 5 Non-daters | 46 (30.3%) | .11 (.82) | .88 (.55) | -1.65 (.49) | .78 (.64) | -.54 (.77) | .72 (.84) |
| Profile 6 Daters | 73 (49.0%) | -.21 (.96) | -1.42 (.53) | -1.47 (.56) | .78 (.46) | -.37 (.83) | .27 (.91) |
| Profile 6 Non-daters | 76 (51.0%) | -.16 (1.00) | -1.37 (.52) | -1.34 (.64) | .80 (.45) | -.58 (.79) | .03 (.95) |
| Profile 7 Daters | 931 (73.4%) | -.31 (.69) | -.19 (.43) | -.25 (.66) | -.39 (.47) | .20 (.70) | **-.20 (.75)** |
| Profile 7 Non-daters | 338 (26.6%) | -.30 (.78) | -.27 (.46) | -.28 (.76) | -.29 (.51) | .18 (.68) | **-.46 (.81)** |
| Profile 8 Daters | 202 (63.5%) | -.77 (.83) | -1.70 (.42) | -.80 (.84) | -.60 (.54) | .75 (.71) | -.54 (.94) |
| Profile 8 Non-daters | 116 (36.5%) | -.89 (.90) | -1.80 (.43) | -.68 (.93) | -.60 (.54) | .58 (.72) | -.79 (.98) |
| Profile 9 Daters | 236 (74.0%) | -.99 (.85) | .09 (.59) | -.92 (.88) | -1.61 (.50) | 1.23 (.55) | -.47 (1.01) |
| Profile 9 Non-daters | 83 (26.0%) | -1.17 (.80) | -.05 (.58) | -.84 (.81) | -1.76 (.51) | 1.11 (.65) | -.79 (1.00) |
| Profile 10 Daters | 123 (65.4%) | -1.67 (.67) | -1.66 (.52) | -1.33 (.82) | -2.08 (.46) | 1.27 (.58) | -1.22 (.82) |
| Profile 10 Non-daters | 65 (34.6%) | -1.55 (.68) | -1.73 (.47) | -1.23 (.88) | -2.00 (.47) | 1.09 (.70) | -1.30 (.84) |

*Note.* **Bold** means indicate there was a statistically significant difference between Daters and Non-daters using 2-tailed independent samples *t*-tests that were Bonferroni-corrected (p<.0007).

**Additional Outcome Measures**

In addition to the measures described in the paper, we assessed the following two measures.

**Loneliness**

Participants completed an 8-item version of the UCLA Loneliness Scale (ULS-8; Hays & DiMatteo, 1987). Examples include “I feel isolated from others” and “I feel left out,” which were rated on a 4-point scale (1=I often feel this way to 4=I never feel this way). Cronbach’s α=.84.

**Depressive Symptomatology**

To assess depressive symptomatology, we used the depression scale from the National Health Interview Survey (Villarroel & Terlizzi, 2020). Participants rated 6 items for how often they felt specific ways (e.g., “hopeless,” “worthless”) during the last 30 days on a 5-point scale (1=*none of the time* to 5=*all of the time*). Cronbach’s α=.92.

**Further Validating the 10 Profiles**

We sought to further validate the profiles by determining whether they were useful when predicting other well-being-related outcomes—specifically, loneliness and depressive symptomatology (see Supplemental Tables S7-S8). Across each profile model, both self-esteem and neuroticism were strong predictors of loneliness and depression. Lower friend and family satisfaction also tended to predict higher loneliness.

**Table S7**

*Loneliness Regression Models*

|  |  | Friend Satisfaction | | Family  Satisfaction | | Self-Esteem | | Neuroticism | | Extraversion | |
| --- | --- | --- | --- | --- | --- | --- | --- | --- | --- | --- | --- |
|  | *R^2^* | *β* | *p* | *β* | *p* | *β* | *p* | *β* | *p* | *β* | *p* |
| Full Sample | .56 | **-.16** | <.001 | **-.13** | <.001 | **-.34** | <.001 | **.30** | <.001 | **-.12** | <.001 |
| Daters | .57 | **-.20** | <.001 | **-.10** | <.001 | **-.32** | <.001 | **.29** | <.001 | **-.13** | <.001 |
| Non-Daters | .56 | **-.12** | <.001 | **-.15** | <.001 | **-.34** | <.001 | **.31** | <.001 | **-.12** | <.001 |
| Profile 1 | .20 | -.12 | .105 | -.09 | .069 | **-.39** | <.001 | **.22** | <.001 | **-.14** | <.001 |
| Profile 2 | .24 | **-.20** | <.001 | **-.12** | .001 | **-.45** | <.001 | **.39** | <.001 | **-.13** | <.001 |
| Profile 3 | .19 | **-.28** | <.001 | -.01 | .745 | **-.23** | <.001 | **.30** | <.001 | **-.13** | <.001 |
| Profile 4 | .28 | -.16 | .395 | **-.39** | .001 | **-.34** | .014 | .20 | .046 | **-.17** | .026 |
| Profile 5 | .32 | **-.35** | .005 | -.20 | .119 | -.19 | .11 | **.40** | <.001 | -.10 | .184 |
| Profile 6 | .32 | **-.33** | .017 | -.08 | .51 | **-.37** | .017 | **.51** | <.001 | -.14 | .082 |
| Profile 7 | .15 | **-.19** | <.001 | **-.13** | <.001 | **-.30** | <.001 | **.27** | <.001 | **-.12** | <.001 |
| Profile 8 | .12 | -.04 | .714 | **-.17** | .001 | **-.31** | <.001 | **.16** | .007 | .00 | .971 |
| Profile 9 | .20 | **-.13** | .023 | **-.18** | <.001 | **-.26** | <.001 | **.19** | .001 | **-.13** | <.001 |
| Profile 10 | .24 | -.07 | .475 | -.05 | .369 | **-.28** | .005 | **.33** | <.001 | -.05 | .362 |

*Note.* Dependent Variable: Loneliness. All regression models controlled for demographics (age, gender, ethnicity, education, and income). **Bold** beta estimates are significant at *p* < .05.

**Table S8**

*Depressive Symptomatology Regression Models*

|  |  | Friend Satisfaction | | Family Satisfaction | | Self-Esteem | | Neuroticism | | Extraversion | |
| --- | --- | --- | --- | --- | --- | --- | --- | --- | --- | --- | --- |
|  | *R^2^* | *β* | *p* | *β* | *p* | *β* | *p* | *β* | *p* | *β* | *p* |
| Full Sample | .60 | -.01 | .454 | **-.08** | <.001 | **-.33** | <.001 | **.44** | <.001 | **.04** | .001 |
| Daters | .59 | -.02 | .086 | **-.10** | <.001 | **-.33** | <.001 | **.43** | <.001 | **.04** | .001 |
| Non-Daters | .59 | .01 | .687 | **-.04** | .016 | **-.32** | <.001 | **.45** | <.001 | .00 | .800 |
| Profile 1 | .31 | **-.16** | .004 | -.04 | .253 | **-.21** | <.001 | **.31** | <.001 | .03 | .249 |
| Profile 2 | .31 | .04 | .274 | -.03 | .198 | **-.14** | <.001 | **.34** | <.001 | .02 | .277 |
| Profile 3 | .33 | -.06 | .367 | .01 | .836 | **-.38** | <.001 | **.50** | <.001 | .01 | .747 |
| Profile 4 | .37 | .12 | .274 | -.08 | .243 | -.09 | .278 | **.35** | <.001 | .01 | .776 |
| Profile 5 | .38 | -.19 | .103 | -.09 | .450 | **-.23** | .042 | **.46** | <.001 | -.12 | .077 |
| Profile 6 | .44 | -.02 | .884 | -.06 | .483 | -.14 | .216 | **.55** | <.001 | .08 | .175 |
| Profile 7 | .29 | .01 | .787 | **-.12** | <.001 | **-.40** | <.001 | **.43** | <.001 | **.06** | .019 |
| Profile 8 | .34 | -.05 | .589 | -.09 | .083 | **-.36** | <.001 | **.57** | <.001 | .07 | .134 |
| Profile 9 | .23 | .02 | .780 | **-.12** | .024 | **-.36** | <.001 | **.48** | <.001 | -.01 | .864 |
| Profile 10 | .38 | -.14 | .219 | **-.16** | .019 | **-.29** | .018 | **.68** | <.001 | .00 | .954 |

*Note.* Dependent Variable: Depressive Symptomatology. Profiles sorted from highest to lowest life satisfaction. All regression models controlled for demographics (age, gender, ethnicity, education, and income). **Bold** beta estimates are significant at *p* < .05.

**Table S9**

*Full Sample Bivariate Correlations*

| Variable | 1 | 2 | 3 | 4 | 5 | 6 | 7 | 8 |
| --- | --- | --- | --- | --- | --- | --- | --- | --- |
| 1. Life Satisfaction | — |  |  |  |  |  |  |  |
| 2. Friend Satisfaction | .45 | — |  |  |  |  |  |  |
| 3. Family Satisfaction | .52 | .41 | — |  |  |  |  |  |
| 4. Self-esteem | .60 | .29 | .37 | — |  |  |  |  |
| 5. Neuroticism | -.40 | -.18 | -.31 | -.59 | — |  |  |  |
| 6. Extraversion | .36 | .42 | .21 | .34 | -.18 | — |  |  |
| 7. Loneliness | -.56 | -.41 | -.44 | -.65 | .59 | -.37 | — |  |
| 8. Depressive Symptomatology | -.44 | -.19 | -.34 | -.65 | .70 | -.17 | .62 | — |

*Note.* All Pearson zero-order bivariate correlations are significant at *p* < .001.

**References**

Brown, A. (2020). *Nearly half of U.S. Adults say dating has gotten harder for most people in the last 10 years*. <https://www.pewresearch.org/social-trends/2020/08/20/nearly-half-of-u-s-adults-say-dating-has-gotten-harder-for-most-people-in-the-last-10-years/>

Gunaydin, G., Oztekin, H., Karabulut, D. H., & Salman-Engin, S. (2021). Minimal social interactions with strangers predict greater subjective well-being. *Journal of Happiness Studies, 22*(4), 1839-1853. <https://doi.org/10.1007/s10902-020-00298-6>

Schroeder, J., Lyons, D., & Epley, N. (2021). Hello, stranger? Pleasant conversations are preceded by concerns about starting one [Advance online publication]. <https://doi.org/10.1037/xge0001118>

Stutzer, A., & Frey, B. S. (2006, 2006/04/01/). Does marriage make people happy, or do happy people get married? *The Journal of Socio-Economics, 35*(2), 326-347. <https://doi.org/10.1016/j.socec.2005.11.043>
